# Social Support, social ties, and cognitive function of women with breast cancer: findings from the Women’s Health Initiative (WHI) Life and Longevity After Cancer (LILAC) Study

**DOI:** 10.1007/s00520-022-07505-5

**Published:** 2022-12-16

**Authors:** Yesol Yang, Eric M. McLaughlin, Michelle J. Naughton, Maryam B. Lustberg, Timiya S. Nolan, Candyce H. Kroenke, Julie C. Weitlauf, Nazmus Saquib, Aladdin H. Shadyab, Shawna Follis, Kathy Pan, Electra D. Paskett

**Affiliations:** 1 Department of Internal Medicine, The Ohio State University Comprehensive Cancer Center-James, 406 W 10thAvenue, Columbus, OH 43210 USA; 2 Center for Biostatistics, The Ohio State University, 1800 Cannon Drive, Columbus, OH 43210 USA; 3 College of Medicine, Department of Internal Medicine, Division of Cancer Prevention and Control, The Ohio State University, 1590 N. High Street, Columbus, OH 43201 USA; 4 Yale Cancer Center, 333 Cedar Street, New Haven, CT 06520 USA; 5 College of Nursing, The Ohio State University, 1585 Neil Avenue, Columbus, OH 43210 USA; 6 Division of Research, Kaiser Permanente Northern California, 2000 Broadway, Oakland, CA 94612 USA; 7 Veterans Affairs Palo Alto Health Care System, 3801 Miranda Avenue, (151Y), Palo Alto, CA USA; 8 College of Medicine, Sulaiman AlRajhi University, Albukairiyah, 51942 Saudi Arabia; 9 Herbert Wertheim School of Public Health and Human Longevity Science, University of California, San Diego, 9500 Gilman Drive #0725, La Jolla, CA 92093 USA; 10 Department of Medicine, Stanford Prevention Research Center, Stanford University, Stanford, CA 94304 USA; 11 Southern California Kaiser Permanente, 9400 Rosecrans Avenue, Bellflower, CA 90706 USA

**Keywords:** Cognitive function, Breast cancer survivors, Social support, Social ties

## Abstract

**Purpose:**

This study examined associations between self-reported cognitive functioning and social support as well as social ties among women with breast cancer.

**Methods:**

The study included 3351 women from the Women’s Health Initiative Life and Longevity After Cancer cohort who were diagnosed with breast cancer stages I–III. Social support was assessed using a modified Medical Outcomes Study (MOS) Social Support Survey, and marital status was obtained from the baseline questionnaire. We also assessed social ties (e.g., number of friends, relatives, living children) and cognitive function (Functional Assessment of Cancer Therapy-Cognitive Function [FACT-COG]) on the year-1-follow up questionnaire. Multivariable quantile regression was used to estimate the changes in median cognitive scores. Kruskal–Wallis tests were used to assess the association of cognitive function with social ties.

**Results:**

The majority of participants were non-Hispanic White (93.3%), presently married (49%), with at least a 4-year college degree (53.2%), and had been diagnosed with localized breast cancer (79%). A 10-point higher social support score correlated to a 0.32 higher (better) median cognitive score (*p* < 0.001). Women who were presently married tended to have better cognition than women who were divorced/separated or widowed (*p* = 0.01). Significant associations were also present for having close relatives (*p* < 0.001) or friends (*p* < 0.001), with cognitive scores being higher in those with at least one close relative or friend compared to none.

**Conclusion:**

Women reporting higher social support and greater numbers of friends or relatives have higher cognitive functioning. Compared to divorced or separated women, married women were likely to have higher cognitive functioning. These findings suggest that social support assessments have the potential to help identify women at higher risk of cognitive decline.

## Introduction

In the USA, approximately 60% of breast cancer survivors are 65 years and older [1]. Compared to younger breast cancer survivors, older breast cancer survivors experience more side effects from cancer treatments and a more complicated recovery due to advanced age and comorbidities [2, 3]. Cognitive changes are the most common complications older survivors experience after treatment [4], and include problems with memory, processing speed, concentration, multitasking, and word retrieval [5]. These cognitive problems significantly impact older survivors’ functional status, level of independence, decision-making capacity, treatment adherence, quality of life, and ultimately their survival [6]. Therefore, investigating factors associated with cognitive functioning in older cancer survivors is crucial to identify targets for treatment.

Social support is defined as the perception and the exchange of social resources between at least two individuals (e.g., family, friends, neighbors, and co-workers) [7]. Compared to their younger counterparts, older breast cancer survivors are more likely to have pre-existing chronic diseases and are more vulnerable to treatment toxicities, thus causing them to experience more distress throughout their survivorship period [8–10]. As a result, they are more likely to require ongoing assistance and have many needs including emotional, informational, and tangible support from family or friends [3, 11, 12]. Research, however, has shown that older survivors have fewer supportive relationships to rely on, compared to younger survivors, due to death or illness in their age cohort [12]. Such unmet social support needs among older survivors can lead to social isolation and loneliness, negatively impacting treatment adherence and illness-management behaviors, and ultimately psychological and physical functioning [12–14]. Poor psychological well-being, and diminished physical functioning commonly correlate with decreased brain health and lower cognitive functioning [15, 16]. Thus, having adequate social support for older survivors may be necessary for maintaining good health and cognitive functioning.

Several non-cancer studies have shown that social support is related to cognitive functioning in healthy older adults. For example, one recent Women’s Health Initiative (WHI) study found that social support was positively associated with the cognitive performance among older adults [17]. Other studies have also reported that higher levels of social support are associated with diminished cognitive decline in older adults [18, 19]. Similarly, older adults who received higher levels of emotional and informational support along with increased contact with family and friends (i.e., social ties) have shown better cognitive function [20]. Several other studies further indicated that having positive social support and social ties is associated with the reduced risk of subsequent neurocognitive illness (e.g., dementia, stroke, or other neurological conditions) among older adults [21–25].

Although the mechanisms underlying the associations between social support and cognitive function remains unknown, prior research offers some potential clues. For example, several studies have suggested that social support may lower risk of intermediate physical and psychological factors, including anxiety, depression, and inflammation [26–28], which may accelerate the risk of cognitive decline [26–28]. Similarly, other work suggests that individuals who build social ties within larger social networks have a lower risk of cognitive decline than those who report weak social ties [18, 19, 29, 30]. Several studies indicate that close social ties buffer against psychological distress, which in turn can decrease the risk of cognitive decline [31, 32].

To date, the association of social support with cognitive functioning has not been examined thoroughly among breast cancer survivors. Furthermore, it is unclear whether or how social ties relate to survivors’ cognitive function. To address these gaps, we examined the associations between social support and cognitive functioning among women with breast cancer in the WHI Life and Longevity After Cancer (LILAC) cohort. We hypothesized that higher social support would be associated with better cognitive functioning. We also explored whether specific social ties were associated with lower cognitive functioning.

## Methods

### Study design and participants

Details of the WHI and the WHI LILAC cohort have been described previously [33, 34]. Briefly, between 1993 and 1998, the WHI recruited postmenopausal women between the ages of 50 and 79 years from 40 clinical centers throughout the USA. Participants were randomized into one or more clinical trials (*n* = 68,132) or an observational study (*n* = 93,676). Participants were followed for up to 10 years within the WHI, and many continued follow-up in the WHI extension studies (including the LILAC study) that began in 2005. In 2013, the WHI LILAC study enrolled WHI participants who had been diagnosed with select cancers (breast, endometrial, ovarian, lung, and colorectal cancers, melanoma, lymphoma, and leukemia) after their enrollment in WHI. The goal of the WHI LILAC was to expand the existing WHI data to support studies of cancer outcomes, survivorship, and molecular epidemiology [35].

For the current analyses, WHI LILAC participants were included if they were diagnosed with breast cancer stage I–III and had complete information on the following variables: age, education, race, marital status, cancer site, cancer stage at diagnosis, self-reported cancer treatment, a symptom checklist, overall cancer worry, physical activity, and social support on the LILAC baseline questionnaire as well as social ties (number of living children, close friends, and close relatives) and cognitive functioning on the year-1 follow-up LILAC questionnaire. All participants in the WHI and the WHI LILAC provided written informed consent before any study activities.

### Social support measure

Social support was measured on the LILAC baseline questionnaire using five survey questions from the Medical Outcome Study (MOS) Social Support Survey [36]. Each question asked how often the respondent believed that social support was available to them when they needed it, e.g., “[How often is someone available] to take you to the doctor if you need to go?; to have a good time with?; to hug you?; to prepare your meals if you are unable to for yourself?; to understand your problems?” Possible responses for each item ranged from 1 (none of time) to 5 (all of the time). Scores were transformed to a 0–100 scale during analyses, with higher scores indicating greater social support. Internal consistency for the score was high (Cronbach *α* > 0.94) and has been validated among cancer survivors [36–38].

### Social ties measures

Social ties were measured by one question about marital status in the baseline LILAC survey and in three survey questions in the 1-year follow up questionnaire. In the baseline questionnaire, participants were asked about their current marital status. The response options were (1) married/living as married, (2) widowed, (3) divorced/separated, and (4) never married. In the 1-year follow up questionnaire, participants were asked: “How many living children do you have?”, “How many relatives do you have with whom you feel close?”, and “How many close friends do you have?”. Possible responses for each item included none, 1–2, 3–5, and 6 or more for living children, and none, 1–2, 3–5, 6–9, and 10 or more for relatives and close friends. We separately analyzed the association of cognitive function with each type of social tie.

### Cognitive functioning measure

Cognitive functioning was assessed on the year-1 LILAC follow-up questionnaire with the 20-item perceived cognitive impairment subscale of the FACT-COG [39]. Participants were asked to rate the frequency of cognitive problems that they had perceived in the past 7 days using a 5-point Likert-type scale. Possible responses for each item ranged from 0 (never) to 4 (several times a day). Answers were reverse coded, and a total score (range 0–80) was calculated. Higher scores indicate better cognitive functioning. Cronbach’s alpha for this subscale ranged from 0.77 to 0.86 [40–42].

### Covariates

Factors that affect cognitive functioning were derived from published literature [43–45] and included age, race, ethnicity, education, cancer stage, self-reported cancer treatment, symptom distress, overall worry, and physical activity. Symptom severity was assessed using 24 items from the WHI symptom checklist, with scores ranging from 0 to 72, with higher scores indicating greater symptom severity. Overall worry was assessed using one survey question, with scores ranging from 1 (not at all) to 10 (a great deal), and a higher score indicating greater worry. Physical activity was derived from the WHI Physical Activity Questionnaire, which measured total duration (minutes/week) of and participation in mild to moderate or strenuous intensities of recreational physical activity [46]. The total minutes of moderate and strenuous exercise per week were calculated for each participant. This measure demonstrated moderate to substantial test–retest reliability [46].

### Statistical analysis

Descriptive statistics were used to summarize the patients’ demographic, clinical and symptom information. Continuous variables were reported as medians (first and third quartiles) and compared with FACT-COG scores using Spearman rank correlations. Categorical variables were reported as counts and percentages, with Fact-COG scores compared using Kruskal–Wallis tests. Linear regression was used to estimate the bivariate relationship between social support and patient cognitive functioning. However, normality assumptions for linear regression were not met by the data, and as a result, quantile regression was used to estimate changes in median FACT-COG scores. Multivariable quantile regression was used to assess whether social support explains the variation in cognitive function, after controlling for the effects of covariates. The final regression model included the primary exposure variable (social support), as well as covariates found to be significantly associated with the outcome of the FACT-COG total score (p < 0.05). Variables in the final regression model include the social support score and covariates (age at diagnosis, minutes of moderate/strenuous exercise per week, symptom count, and worry). Interactions with the social support score were checked for each covariate in the final model using Wald tests, and no interactions were significant. In addition, we conducted stratified analysis for the association of social support with FACT-COG scores in various demographic and clinical subgroups. Interaction effects were tested to evaluate whether the association between social support and FACT-COG scores were modified by race/ethnicity (Non-White or White), age (< 60, 60–69, 70–79, or > 80), marital status (never married, divorced/separated, widowed, or presently married), cancer stage (local or regional), and cancer treatments (chemotherapy, radiation, hormone, or others). Models included the main effects of social support and the variable of interest, along with the interaction term.

As an exploratory analysis, Kruskal–Wallis tests were used to assess the association of cognitive function with social ties (marital status and numbers of living children, close friends, and close relatives). Missing data were few (approximately 7% had missing data for variables in the multivariable quantile regression model), so complete case analysis was used for the multivariable modeling. We also conducted a sensitivity analysis with data imputation but there were no changes from the complete case results. All statistical analyses were performed using SAS version 9.4 (SAS Institute, Inc., Cary, NC).

## Results

### Sample characteristics

Among 3351 women included in the study cohort, the mean (SD) age was 70.2 (SD = 7.5) years. The average time between cancer diagnosis and FACT-COG completion was 9.4 (SD = 5.0) years. The majority of women were Non-Hispanic White (93.3%), presently married (49%), had at least a 4-year college degree (53.2%) and had localized breast cancer (79%). Approximately 70% did not receive chemotherapy but received radiation therapy, and nearly 67% received hormone therapy. Tables 1 and 2 displays characteristics of the study participants, and their correlations with cognitive functioning (FACT-COG). Better cognitive functioning was noted among women who were younger when diagnosed with breast cancer (*r* =  − 0.084, *p* < 0.01), more engaged in moderate/strenuous exercise (*r* = 0.067, *p* < 0.01), and reported lower symptom severity (count: *r* =  − 0.484, *p* < 0.01; severity: *r* =  − 0.486, *p* < 0.01) as well as lower level of worry (*r* = -0.200, *p* < 0.01). However, no association was found between FACT-COG scores and race, ethnicity, education, cancer stage, or type of cancer treatments.

**Table 1:** Kruskal–Wallis test for the association between sample characteristics with FACT-COG scores

Categorical variable		*N* = 3351	FACT-Cognition Score	*P*-value
		*n* (%)	Median [Q1–Q3]	
Race/ethnicity				
American Indian		8 (0.2)	71.5 [62.0–78.5]	0.51
Asian/Pacific Islander		67 (2.0)	73.0 [65.0–76.0]	
Black		129 (3.9)	69.0 [62.0–76.0]	
Hispanic/Latina		11 (0.3)	70.0 [63.0–77.0]	
Other		10 (0.3)	64.5 [57.0–75.0]	
White		3126 (93.3)	70.0 [61.0–76.0]	
Education				
High school diploma, GED, or less		472 (14.2)	69.0 [60.0–76.0]	0.11
Some college, associate degree, training/vocational school		1087 (32.6)	70.0 [61.0–75.0]	
College grad, baccalaureate degree		479 (14.4)	71.0 [62.0–76.0]	
Some post grad, Master’s or Doctoral degree		1293 (38.8)	71.0 [62.0–75.8]	
Cancer stage				
Local		2644 (78.9)	70.0 [61.0–76.0]	0.84
Regional		707 (21.1)	70.0 [62.0–76.0]	
Self-reported cancer treatment				
Chemotherapy	Yes	960 (28.7)	70.0 [61.1–76.0]	0.88
	No	2391 (71.3)	70.0 [61.1–76.0]	
Radiation	Yes	2344 (70.0)	70.0 [62.0–76.0]	0.34
	No	1007 (30.0)	70.0 [60.0–76.0]	
Hormone	Yes	2256 (67.3)	70.3 [62.0–76.0]	0.15
	No	1095 (32.7)	70.0 [61.0–75.8]	
Other (tumor vaccine, stem cell transplantation)	Yes	172 (5.1)	69.2 [59.0–74.3]	0.06
	No	3179 (94.9)	70.0 [61.3–76.0]

**Table 2:** Spearman rank correlation of sample characteristics with FACT-COG scores

Continuous variable	*N*	Median [Q1–Q3]	Coefficient (*P*-value)
Age at diagnosis (years)	3350	70 [65–75]	− 0.084 (*p* < 0.001)
Minutes of moderate/strenuous exercise per week	3282	0 [0–100]	0.067 (*p* = 0.001)
Symptom checklist count (0–24)	3329	8 [6–11]	− 0.484 (*p* < 0.001)
Symptom severity (0–72)	3329	10 [6–16]	− 0.486 (*p* < 0.001)
Overall worry (0–10)	3325	1 [1–1]	− 0.200 (*p* < 0.001)

### Primary analysis: association between social support and cognitive function

Results for the multivariable regression model are presented in Table 3. In this model, the symptom severity score was not included due to collinearity with symptom count. After adjusting for covariates (age at diagnosis, minutes of moderate/strenuous exercise per week, symptom count, and worry), a 10-point higher social support score corresponded to a 0.32 higher median FACT-COG score (*β* = 0.32, 95% CI: 0.16–0.47, *p* < 0.001). In addition, FACT-COG scores tended to decrease with increasing age at diagnosis ($\beta$ = − 1.07, *p* < 0.001), increasing symptom counts ($\beta$= − 1.20, *p* < 0.001), and increasing worry ($\beta$ = − 1.16, *p* < 0.001). There was no significant association between moderate/strenuous exercise and FACT-COG scores (*p* = 0.41).

**Table 3:** Quantile regression of social support on median FACT-COG scores

	Parameterestimate	95% confidence interval	*P*-value
Adjusted			
Model intercept	86.07	82.70 to 89.45	< 0.001
Primary exposure: Social support (10-point increase)	0.32	0.16 to 0.47	< 0.001
Covariates: Age at diagnosis (10-year increase)	− 1.07	− 1.49 to − 0.65	< 0.001
Minutes per week of moderate/strenuous exercise (60-min increase)	− 0.10	− 0.35 to 0.14	0.41
Symptom count (1 symptom increase)	− 1.20	− 1.29 to − 1.11	< 0.001
Worry interferes w/ daily activities scale (1-point increase)	− 1.16	− 1.76 to − 0.56	< 0.001

Results of the effect modification analyses are presented in Table 4. In these models, we noted that the subgroups of non-white women (American Indian, Asian/Pacific Islander, Black, Hispanic/Latina, Other), women age < 60 at diagnosis, and women who were never married showed insignificant associations between social support and FACT-COG scores, although these groups all had small sample sizes relative to the other subgroups. The associations remained statistically significant for all other subgroups (all *p* < 0.05). While there were some differences in the parameter estimates across the subgroups, no variables (race/ethnicity, age at diagnosis, marital status, cancer stage, self-reported cancer treatments) had a significant modification of the effect of social support on FACT-COG scores (interaction p values all > 0.05).

**Table 4:** Stratified analysis for the association of social support with FACT-COG score in various demographic and clinical subgroups

Categorical variables		*N* = 3351	Estimates for a 10-point increase in social support score		
		*n* (%)	Parameter estimate (95% CI)	Subgroup *P*-value	Interaction *P*-value
Race/ethnicity					
American Indian		8 (0.2)	1.00 (− 8.17 to 10.17)	0.83	0.47
Asian/Pacific Islander		67 (2.0)	− 0.36 (− 1.58 to 0.85)	0.56	
Black		129 (3.9)	1.00 (− 0.04 to 2.04)	0.06	
Hispanic/Latina		11 (0.3)	0.57 (− 25.38 to 26.53)	0.97	
Other		10 (0.3)	2.55 (− 1.33 to 6.42)	0.20	
White		3126 (93.3)	0.83 (0.61 to 1.06)	< 0.001	
Age at diagnosis					
< 60		283 (8.5)	0.60 (− 0.21 to 1.41)	0.15	0.50
60–69		1275 (38.1)	0.80 (0.48 to 1.12)	< 0.001	
70–79		1406 (42.0)	0.86 (0.47 to 1.24)	< 0.001	
80 +		387 (11.6)	1.28 (0.59 to 1.96)	< 0.001	
Marital status					
Never married		159 (4.7)	0.40 (− 0.53 to 1.33)	0.40	0.38
Divorced/separated		439 (13.1)	0.67 (0.18 to 1.15)	0.007	
Widowed		1094 (32.7)	1.06 (0.63 to 1.48)	< 0.001	
Presently married		1642 (49.0)	1.08 (0.71 to 1.44)	< 0.001	
Cancer stage					
Local		2644 (78.9)	0.88 (0.63 to 1.14)	< 0.001	0.89
Regional		707 (21.1)	0.92 (0.46 to 1.38)	< 0.001	
Self-reported cancer treatment					
Chemotherapy	Yes	960 (28.7)	0.91 (0.49 to 1.33)	< 0.001	0.65
	No	2391 (71.3)	0.80 (0.54 to 1.06)	< 0.001	
Radiation	Yes	2344 (70.0)	0.86 (0.61 to 1.12)	< 0.001	0.79
	No	1007 (30.0)	0.80 (0.35 to 1.24)	< 0.001	
Hormone	Yes	2256 (67.3)	0.91 (0.65 to 1.17)	< 0.001	0.52
	No	1095 (32.7)	0.75 (0.30 to 1.20)	0.001	
Other (tumor vaccine, stem cell transplantation)	Yes	172 (5.1)	1.43 (0.40 to 2.46)	0.007	0.26
	No	3179 (94.9)	0.88 (0.67 to 1.10)	< 0.001	

### Exploratory analysis*:* association between social tie variables and cognitive function

Table 5 shows that FACT-COG scores were significantly associated with marital status (*p* = 0.01), number of children (*p* = 0.01), number of relatives (*p* < 0.001), and number of friends (*p* < 0.001). Cognitive scores tended to be lower among those with more living children (median = 71 for none; median = 66 for 6 or more), but were higher with more relatives (median = 70 for none; median = 73 for 10 or more) or friends (median = 69 for none; median = 72 for 10 or more). Women who were presently married (median = 71) or never married (median = 72) had higher median cognitive scores, while women who were widowed had the lowest scores (Median = 69).

**Table 5:** Kruskal–Wallis test for the association of social ties with medial FACT-COG scores

Social tie variables	*N* = 3351	FACT-cognition score	*P*-value
	*n* (%)	Median [Q1–Q3]	*P*-value
Marital status			
Never married	159 (4.7)	72.0 [63.0–77.0]	0.01
Divorced/separated	439 (13.1)	70.0 [62.0–76.0]	
Widowed	1094 (32.7)	69.0 [60.0–75.0]	
Presently married	1642 (49.0)	71.0 [62.0–76.0]	
Number of living children			
None	410 (12.5)	71.0 [62.1–76.0]	0.01
1–2	1331 (40.7)	71.0 [62.0–76.0]	
3–5	1409 (43.1)	70.0 [61.0–75.8]	
6 or more	121 (3.7)	66.0 [58.0–74.0]	
Number of close relatives			
None	305 (9.4)	70.0 [62.0–76.0]	< 0.001
1–2	1019 (31.5)	70.0 [61.0–75.0]	
3–5	1010 (31.3)	70.0 [61.1–75.0]	
6–9	503 (15.6)	71.0 [62.0–76.0]	
10 or more	395 (12.2)	73.0 [64.0–77.0]	
Number of close friends			
None	61 (1.9)	69.0 [51.0–77.0]	< 0.001
1–2	513 (15.8)	69.0 [60.0–75.0]	
3–5	1319 (40.6)	70.0 [61.0–75.0]	
6–9	790 (24.3)	72.0 [63.0–76.0]	
10 or more	567 (17.5)	72.0 [62.4–77.0]	

## Discussion

To our knowledge, this is one of the few studies to examine the association of social support with cognitive functioning among a sample of US older female breast cancer survivors. In support of our hypothesis, our findings indicate that survivors with higher social support demonstrated higher perceived cognitive functioning than those women with lower social support. We also found that perceived cognitive functioning was significantly associated with marital status, and the number of living children, relatives and friends.

Our findings are in line with previous studies. Past research has found that social support is associated with better cognitive functioning in older adults[17, 19, 47], and is protective against memory decline [18]. One possible explanation for these associations is that social support can alleviate psychological distress, leading to improved cognitive function (or fewer declines) [48]. Similarly, several studies found that social support decreases the levels of psychological distress (anxiety, depression) among breast cancer patients[49, 50]. Future studies should include repeated measures of cognitive functioning and social support to further explore the dynamics of social support and cognitive function in breast cancer survivors, along with mechanisms that might explain this association.

These study findings also contribute to the increasing literature focused on the association of cognitive functioning with social relationships (e.g., social support and social ties). Recent systematic reviews have reported that older populations showed a greater decline in their cognition when their social relationships were functionally (e.g., social support) and structurally (e.g., social ties) poor [47, 51]. This previous research is consistent with our finding that cognitive functioning is associated with structural aspects (e.g., marital status and the number of friends, relatives, or children) as well as functional aspects (e.g., social support) of social relationships. In contrast to association of cognitive function with the number of friends and relatives, survivors with more than six living children showed lower levels of cognitive function compared to those with no children in this current study. A possible explanation for this negative association is that conflicts that occur in larger size family can lead to increased emotional distress [52, 53], resulting in cognitive decline [54]. Another possible explanation can be that women who have undergone childbirth more often may have relatively poor health conditions (e.g., changes in blood lipids and blood pressure, insulin resistance, weight gain) during and after pregnancy than those who have fewer births; in turn, those with poor health conditions may have poorer cognitive health later in life [55–58]. Further studies need to include more comprehensive aspects of social factors that may be associated with the cognitive function of breast cancer survivors. Increased understanding of this association will help identify survivors at risk of cognitive decline and assist in developing prevention and early intervention strategies for those with cognitive problems.

The findings of this study highlight the importance of social support among older adults with breast cancer. Healthcare providers need to encourage older survivors to participate in social activities to build supportive social relationships in order to improve or maintain overall health and cognitive functioning. It is important to inform survivors of how to ask for social support. Doing so will help buffer the impacts of cancer and cancer treatments and improve overall survivorship experiences. It is also important to educate survivors’ informal social networks (e.g., family or friends) about the positive impacts of support and survivors’ difficulties in asking for needed social support [3, 59]. Thus, in order to provide adequate social support to older cancer survivors, more research on social support interventions is needed.

The strengths of this study include the large number of study participants with extensive demographic, psychosocial, and cancer diagnosis and treatment data. However, this study has several limitations. First, social support, social ties, and cognitive functioning were only measured at one time point in the LILAC cohort, so we could not investigate a causal relationship between social support and cognitive function. Second, the social support measure included in LILAC provided limited data on the types of social support (e.g., tangible assistance, esteem support, network support). Future studies are needed that include data on the types of social support and additional social factors (e.g., social activity or social integration) to better understand if (and how) cognitive functioning is associated with these variables. Third, the included study participants were predominantly Non-Hispanic White (93.3%). This could limit the generalizability of these findings to other racial and ethnic groups. Future studies are needed that include participants with diverse racial/ethnic backgrounds. Lastly, this study used one subscale of the FACT-COG. Thus, we were unable to determine whether the obtained cognitive scores were clinically meaningful, because the published values are derived from the FACT-COG total score. [60, 61] Future studies that include full batteries of FACT-COG would help strengthen these study findings.

## Conclusion

In summary, among post-menopausal older women with breast cancer, those with higher social support had better cognitive functioning than those with lower social support. Compared to divorced/separated women, married women were likely to have higher cognitive functioning. Also, those with a greater number of close friends or relatives had better cognitive functioning than those with no or one friend or relative. These findings suggest that provision of social support and availability of social ties during a time when women are at risk of social isolation could help prevent cognitive decline in aging breast cancer survivors. The current research contributes to the existing literature that suggests the importance of social support and social ties as factors associated with cognitive health.

## Data Availability

The datasets during and/or analyzed during the current study are available from the corresponding author on reasonable request.

## Abbreviations

LILAC: Life and Longevity After Cancer cohort
WHI: Women’s Health Initiative
